# Efficacy of surgical excision and sub-dermal injection of triamcinolone acetonide for treatment of keloid scars after caesarean section: a single blind randomised controlled trial protocol

**DOI:** 10.1186/s13063-019-3465-6

**Published:** 2019-06-18

**Authors:** Seng Chai Chua, Beata Gidaszewski, Marjan Khajehei

**Affiliations:** 1 Department of Obstetrics and Gynaecology, Westmead Hospital, Sydney, NSW Australia; 2 Department of Women’s and Newborn Health, Westmead Hospital, Sydney, Australia; 3 The University of Sydney, Sydney, Australia; 4 University of New South Wales, Room 3046, Research and Education Network (REN) Building, Westmead Public Hospital, Hawkesbury Rd, Westmead, Sydney, NSW 2145 Australia

**Keywords:** Keloid, Scar, Caesarean section, Triamcinolone acetonide, Injection

## Abstract

**Background:**

One of the first-line options to treat keloid scars is corticosteroid injection after excision of the existing scar. A thorough literature search has shown a lack of research on the injection of corticosteroid injection immediately after the excision of the existing caesarean section keloid scars. Therefore, in the proposed study, we aim to investigate the effect of surgical excision and corticosteroid (triamcinolone acetonide) injection immediately after surgical removal of old caesarean section keloid scars on the recurrence of the scars. This is a protocol for a randomised controlled trial.

**Methods/design:**

Pregnant women (*n* = 150), who attend antenatal clinics at Westmead Hospital in New South Wales, Australia, have a keloid scar from a previous caesarean section, meet the inclusion criteria and sign the consent form, will be randomised to either the control or the intervention group. The control group will receive surgical excision of the keloid scar at the beginning of the procedure during skin incision. The baby will be delivered according to normal procedures, and routine wound closure will be performed in accordance with National Institute for Health and Care Excellence guidelines. The intervention group will receive surgical excision of the keloid scar after the delivery of the baby, and closure of the uterus layers, rectus sheath and the fat layer will be completed as explained above. Then, triamcinolone acetone will be injected sub-dermally at the time of wound closure. Two ampules of triamcinolone acetonide will be administered at a single dose; each ampule contains 10 mg/1 ml active medication. The surgeon will inject one ampule along the entire length of the upper edge of the skin incision and one ampule along the entire length of the lower edge of the skin incision, using a 25 G needle. After the procedure is completed, the surgeon will fill in the post-operation survey.

The participants will be followed up post-operation, daily on the ward and then at 6 weeks, 6 months and 12 months post-partum. Main outcomes are (1) keloid formation after caesarean section and (2) changes in the appearance and specification of the keloid scar after the intervention.

**Discussion:**

We anticipate that surgical excision and steroid injection will be a safe, lasting and cost-effective treatment in the management of caesarean keloid scars which will be useful for patients unable to undergo cosmetic surgery due to clinical or financial reasons.

**Trial registration:**

Australian New Zealand Clinical Trials Registry, ACTRN12618000984291. Registered on 12 June 2018.

**Electronic supplementary material:**

The online version of this article (10.1186/s13063-019-3465-6) contains supplementary material, which is available to authorized users.

## Background

Keloid scars affect 10–15% of all wounds and represent an exaggerated healing response that poses a challenge for physicians. They can appear anywhere on the body and can last for years after the initial injury [1]. Patients at risk of keloid scars are usually younger than 30 years and have darker skin. Darkly pigmented skin is the primary risk factor for keloids, which has a 15–20 fold increased risk due to melanocyte-stimulating hormone anomalies. Black, Hispanic and Asian persons are more likely to develop keloid scars than Caucasians. Keloid scars are more than just disfiguring cosmetically; many are also pruritic and painful. They also often result in severe emotional distress [2].

Keloid scars are associated with deteriorated mental health during the post-natal period and can contribute to negative body image, impaired intimate relationships and symptoms of depression. Patients with keloid scars have been reported to be unhappy with their scars due to their perceived stigma and psychological association [3–6]. Consequently, this can result in these patients being unsociable and may interfere with their communication skills, personal relationships and work, life and leisure activities. Patients are usually concerned about the diagnosis and persistent nature of the scars and report that non-empathic management by general physicians and frustrations of current treatment compound their distress [6].

Any kind of trauma that can reach the reticular dermis and cause cutaneous injury (such as insect bites, burns, surgery, vaccinations, skin piercings, acne, folliculitis, etc.) can create abnormal wound healing and trigger keloid scar formation in predisposed people. The abnormal wound healing is described by continuous and histologically localised inflammation and increased inflammatory cells, which result in the increase of fibroblasts, proinflammatory factors, newly made blood vessels and collagen deposits. These changes can stimulate chronic inflammation, which in turn causes the invasive growth of keloid scars. In addition, the upregulation of proinflammatory factors in pathological scars suggests that keloid scars are inflammatory conditions of the reticular dermis of the skin [7, 8].

Based on the cellular mechanism of keloid formation, one of the first-line options to treat keloid scars is corticosteroid injection into the affected area. The steroids have both direct anti-inflammatory and vasoconstrictive effects. Administration of corticosteroids results in the whitening of keloid scars, suggesting a decrease of blood flow in the scar due to vasoconstriction [7].

Another suggested treatment of keloid scars is surgical removal of the scar. However, since surgical removal of the keloid usually only provides temporary cosmetic relief and invariability followed by even more aggressive regrowth of scar tissue in 50–100% of cases, many clinicians perform surgical excision of the keloid scar followed by sub-dermal corticosteroid injections. This has been suggested to be effective for patients with small or single site lesions [9].

Some studies have used immediate wound edge corticosteroid injection after the excision in patients with hypertrophic scars and keloid scars, followed by regular injection of corticosteroid: usually a weekly injection for 2–5 weeks followed by monthly injections for 3–6 months [10]. This combination of surgical removal and corticosteroid injections has been shown to improve outcome. This dose of steroid is substantially more than the stat dose we plan to use in our study and requires multiple visits and repeated injections.

A thorough literature search has shown a lack of research on the surgical excision and injection of corticosteroid injection immediately after the excision of the existing caesarean section keloid scars. Therefore, in the proposed study, we aim to investigate the effect of corticosteroid (triamcinolone acetonide) injection immediately after surgical removal of old caesarean section keloid scars on the recurrence of the scars.

### Hypothesis

Given that triamcinolone acetonide has been used for treatment of keloid scar in other areas of the body [11, 12], we hypothesise that excision of a previous caesarean section keloid scar in conjunction with sub-dermal injection of triamcinolone acetonide will reduce the recurrence and formation of the keloid scar. We would also expect positive patient satisfaction from the treatment and a decline in symptoms of post-partum depression.

### Composite primary objective

A primary objective is to determine if surgical excision and sub-dermal injection of triamcinolone acetonide at the time of subsequent caesarean section is effective in preventing or reducing the amount of keloid formation in patients with a previous history of keloid scar after caesarean section. We also aim to investigate whether the appearance and specification of the keloid scar changes after the intervention (type of response).

### Secondary objectives

Secondary objectives of the trial are to:

- Evaluate the effect of surgical excision and keloid scar treatment on symptoms of depression in women with keloid scars
- Determine whether surgical excision and sub-dermal injection of triamcinolone acetonide at the time of caesarean section changes the appearance (width, length, height) and specification (redness, hardness and elevation of the scar compared to the surrounding skin as well as discomfort including itching, pain and swelling) of the keloid scar
- Assess side effects of the surgical excision and sub-dermal injection of triamcinolone acetonide at the time of caesarean section
- Investigate patients’ satisfaction with the treatment and their perception with the changes in their keloid scar after surgical excision and sub-dermal injection of triamcinolone acetonide at the time of caesarean section.

## Methods/design

### Setting

**Fig. 1:**
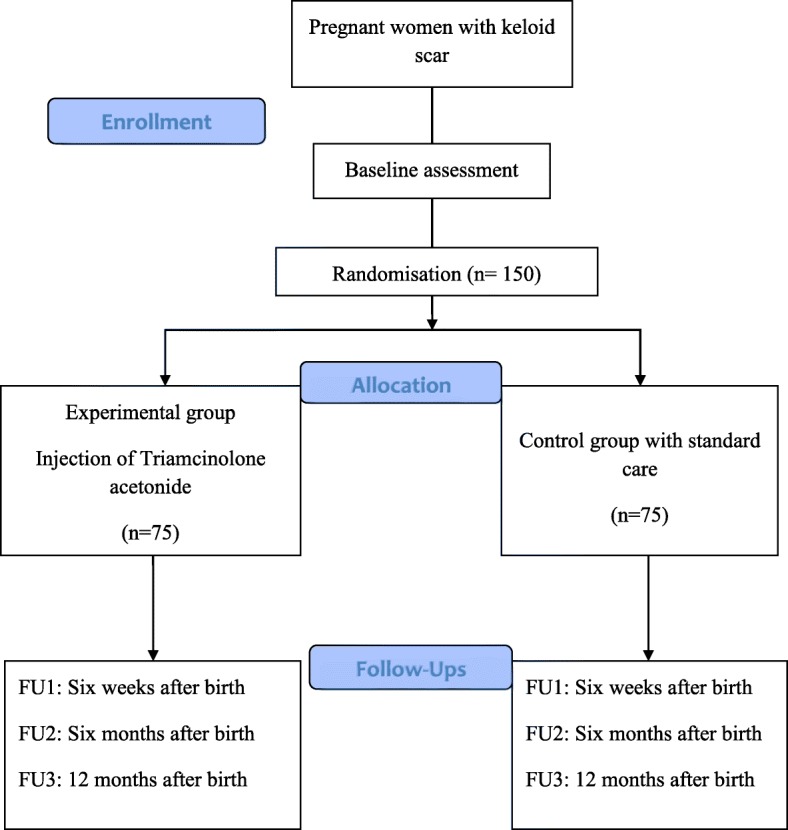
Flow diagram of the trial

The patients will be recruited from the antenatal clinics that are booked for delivery at Westmead Hospital, a large tertiary unit serving a multiethnic, low-middle income population in addition to referred high-risk pregnancies in Western Sydney, New South Wales, Australia. Figure 1 is a flow diagram of the trial. The Standard Protocol Items: Recommendations for Interventional Trials (SPIRIT) checklist is provided as Additional file 1.

### Study design

This will be a superiority randomised controlled clinical trial with parallel intervention assignment.

### Ethical approval

The study protocol, amendments and consent form were approved by the Western Sydney Local Health District Human Research Ethics Committee.

### Participants

The inclusion criteria for the study are (1) age between18–45 years old; (2) being pregnant at the time of recruitment; (3) presence of a keloid scar from a previous caesarean section; (4) planned caesarean section for the current pregnancy. Women of all ethnicities with a keloid scar following a previous caesarean section will be eligible to participate in the study.

The exclusion criteria for the study are (1) being primigravida and (2) having a previous caesarean section with no keloid scar. Since the aim of the study is to investigate the impact of treatment on the caesarean section keloid scar, the type of skin excision in the caesarean section (vertical or horizontal) will not be an inclusion/exclusion criterion.

### Sample size

Using the estimates from the study by Khalid et al. [13], the intervention will increase the incidence of efficacy of treatment for keloid scarring from 45% in the excision followed by radiation group to 70% in the excision followed by intralesional triamcinolone acetonide and 5-fluorouracil group. We calculated that a total of 150 patients (75 in each group) would provide 80% power with a two-sided significance level of 5% to detect this difference between the groups. This allows for 10% non-compliance.

### Composite primary outcome

The primary outcomes are:

- Reduction in the amount of keloid formation after the intervention in patients with a previous history of keloid caesarean scar
- Changes in the appearance and specification of the keloid scar after the intervention.

The baseline information including measurement of the scar will be collected at the time of consent and/or around 36 weeks when the patient is consented for the surgery. The scar will be photographed and measured for length and width in millimetres; elevation, hardness and erythema will be graded by the assessor on a 3-point scale (0 = none, 1 = partial, 2 = along entire scar). A subjective symptom score will be graded by the patient for pruritus, pain and swelling on a 3-point scale (0 = none, 1 = occasionally, 2 = all the time).

### Secondary outcomes

The secondary outcomes are:

- Percentage of women with depression, anxiety and stress
- Patients’ satisfaction with the treatment and changes in their keloid scars.

### Outcome measures

#### Data collection sheet

A purposefully designed questionnaire consisting of two sections will be used to collect demographics (including age, country of birth, ethnic background, weight, height, BMI, number of previous vaginal births and/or caesarean sections, number of previous miscarriages, abortion and stillbirth, family history of keloid scars, history of any previous surgery and keloid scar on another site of body, gestational age at the time of baseline assessment) and any prior treatment of the keloid scar (section 1) and data on the keloid scar (section 2) including redness, hardness and elevation of the scar compared to the surrounding skin (0 = none, 1 = partially, 2 = entire scar). This section of the questionnaire also enquires about subjective symptoms of the scar including, but not limited to, itching, pain and swelling (0 = none, 1 = occasionally, 2 = all the time). The scar will be measured in millimetres using a hardened stainless steel 150mm Digital Vernier Caliper (Kincrome Pty Ltd, Scoresby, Australia) with a liquid-crystal display (LCD) screen to read an accurate measure to the nearest 0.02 mm. We will also take photographs of the scar to compare the visual changes. Section 2 of the questionnaire will be used at follow-up visits to collect data on the changes in the keloid scar. The same 150mm Digital Vernier Caliper will be used at follow-up visits, and the scars will be photographed again. To improve the objective validity of the trial and minimise recall bias of patients in the symptom scores, the participants will be shown photographs of the scar both before and one year after the intervention. They will be asked if they see any improvement before and after participating in the trial, both before and after seeing their scar photographs. In addition, the participants will assess and grade their satisfaction with the treatment on a 4-point scale (0 = no improvement/poor, 1 = fair, 2 = good, 3 = excellent). The participants will be queried on their overall satisfaction with the treatment (5 = very satisfied, 4 = slightly satisfied, 3 = neutral satisfaction, 2 = slightly unsatisfied, 1 = very unsatisfied). They will also be asked if they think they were in the intervention arm or the control arm of the trial before revealing this information to them at the final visit.

#### Depression, Anxiety and Stress Scale-21 (DASS-21)

The DASS-21 is the short version of the DASS-42 questionnaire. It contains a set of three self-report scales and will be used to measure anxiety, depression and stress during pregnancy and after childbirth. The DASS-21 has been shown to have high internal consistency and to yield meaningful discriminations in a variety of settings. It should meet the needs of both researchers and clinicians who wish to measure current state or change in state over time on the three dimensions of depression, anxiety and stress. The tool has been used in previous studies on perinatal women in Australia, Canada, Chile, England, Germany, Iceland, New Zealand and the USA with reasonable response rates [14, 15]. Each of the three DASS-21 scales contains 7 items. The Depression scale assesses dysphoria, hopelessness, devaluation of life, self-deprecation, lack of interest/involvement, anhedonia and inertia. The Anxiety scale assesses autonomic arousal, skeletal muscle effects, situational anxiety and subjective experience of anxious affect. The Stress scale is sensitive to levels of chronic non-specific arousal. It assesses difficulty relaxing, nervous arousal and being easily upset/agitated, irritable/over-reactive and impatient. Respondents are asked to use 4-point severity/frequency scales to rate the extent to which they have experienced each state over the past week. Scores for Depression, Anxiety and Stress are calculated by summing the scores for the relevant items and then multiplying each scale score by 2. Then, each score can be transferred to the DASS profile sheet, enabling comparisons to be made between the three scales and also giving percentile rankings and severity labels as shown in the following table.

**Table:** 

	Depression	Anxiety	Stress
Normal	0–9	0–7	0–14
Mild	10–13	8–9	15–18
Moderate	14–20	10–14	19–25
Severe	21–27	15–19	26–33
Extremely severe	28+	20+	34+

#### Post-operation survey for the surgeon

After the participant has had her caesarean section, the surgeon will complete a questionnaire. In both arms, the surgeon will be asked if the keloid scar was completely excised, and any difficulty of the ascribed surgical technique will be noted. In the intervention arm, the surgeon will also note any difficulty with sub-dermal injection of triamcinolone acetonide, any spill of the medication out of the wound and whether the needle punctured the skin when injecting with triamcinolone acetonide. A diagram is given to indicate the area of anticipated problems if any.

### Blinding

The participants receiving the treatment, the researcher assessing the outcomes and the statistician analysing the data will be blinded to the intervention allocation. The nature of the study is such that the surgeon cannot be blinded to the type of intervention.

### Allocation concealment

Allocation concealment will be used to prevent selection bias by concealing the allocation sequence. This will be done by an independent researcher who works neither in the operating theatre nor in the clinic. This will prevent the researcher from influencing which participants are assigned to a given intervention group and reporting bias.

### Randomisation

Through a parallel intervention assignment, the independent researcher will use a computer-Vgenerated random-numbers list to determine group allocation. The researcher will then place each allocation into sequentially numbered opaque envelopes. There will be permuted blocks of 10 with 1:1 allocation, containing 5 intervention and 5 control allocations in each block. This will help achieve balance across the two groups.

For those who are randomised to triamcinolone acetonide, the independent researcher will insert the medication inside the envelope and, after sealing it, will store the envelope in a locked cabinet inside the medication room in the birth unit. The surgeon who is rostered on the day of the caesarean section will be informed of the recruited participant and will be instructed on where to collect the concealed envelope. All participants will have their randomisation number indicated in the hospital electronic record at the time of study allocation. This is done to inform the researcher assessing the outcomes of the recruiting of the participant and the allocated number, but not the allocated intervention.

### Procedures

All eligible women will be approached at the time of booking into the clinic for caesarean section at around 36 weeks and will be given information about the study. Those women who meet the inclusion criteria and sign the informed consent form will be randomised to either the intervention or the control group by the independent researcher as explained previously (Fig. 1). On the day of the caesarean section, the sealed envelope containing instructions for the type of treatment will be picked up by the surgeon. The surgeon opens the envelope when the woman is ready for the caesarean section.

The control group will receive surgical excision of the keloid scar at the beginning of the procedure during skin incision. The baby will be delivered as normal, and routine wound closure will be performed in accordance with National Institute for Health and Care Excellence (NICE) guidelines as follows: The uterus is closed in two layers followed by closure of the rectus sheath, all using continuous sutures with 1-Vicryl (Johnson & Johnson). The fat layer is closed with interrupted plain gut sutures 2 cm apart. The skin layer is then closed with 3-0 Monocryl (Ethicon) in a sub-cuticular fashion.

The intervention group will receive surgical excision of the keloid scar after the delivery of the baby. Closure of the uterus layers, rectus sheath and the fat layer are completed as explained above. Then, triamcinolone acetone will be injected sub-dermally at the time of wound closure. Two ampules of triamcinolone acetonide (Kenacort®-A 10 suspension, Aspen Pharma Pty Ltd) will be administered at a single dose; each ampule contains 10 mg/1 ml active medication. The surgeon will inject one ampule along the entire length of the upper edge of the skin incision and one ampule along the entire length of the lower edge of the skin incision, using a 25 G needle.

After the procedure is completed, the surgeon will fill in the post-operation survey. The excision of the scar and administration of the injection will be performed by the surgeon who is scheduled to perform the surgery on that day. All the surgeons in the Department of Women’s and Newborn Health will be trained on how to perform the excision and injection of the triamcinolone. The Principal Investigator of the study will assess the fidelity to the intervention by supervising procedures.

### Follow-up

The follow-up period consists of 12 months face-to-face visits (Fig. 1). Post-operation, the patients will be visited daily on the ward for any signs of potential infectious local complications of the treatment and immediate side effects of the medication injection until the discharge home. The patients will then be visited in the women’s health clinic at the hospital for their 6th week post-partum check. The next follow-up times will be 6 months and 12 months post caesarean section. At each follow-up visit, the same assessment and scoring scales will be used to evaluate the changes to the keloid scar. Late side effects of the medication injection as well as symptoms of depression, anxiety and stress, and participants’ satisfaction will be assessed during follow-up visits at 6 weeks, 6 months and 12 months post-partum. The participants will also be asked if they would recommend such a procedure to another woman with a caesarean section keloid scar (yes/no) and if they would repeat the experience again in a future pregnancy (yes/no).

**Fig. 2:**
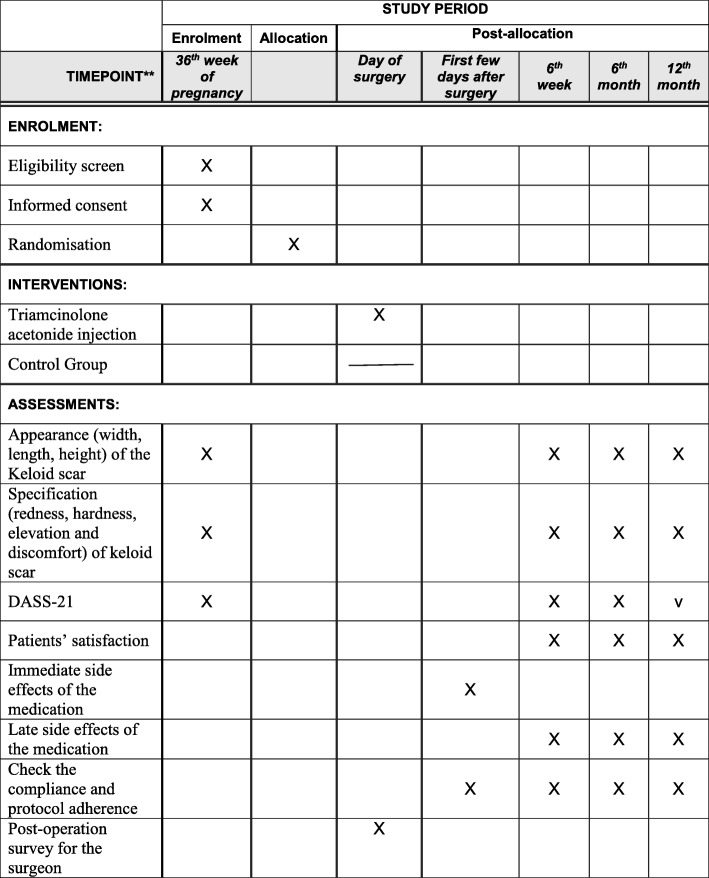
Schedule of enrolment, interventions and assessments

All measurements at baseline and follow-up visits will be performed by the same researcher, who will also make all efforts to ensure compliance with follow-up. As part of continuous clinical care, patients with any residue keloid scar with be given information on further treatment including referral to plastic surgeons if they are interested (Fig. 2).

### Data analysis

Intention-to-treat analysis will be performed using SPSS Advanced Statistics version 24.0 (SPSS, Chicago, IL, USA). Descriptive statistics will be used to summarise the demographic data; binary outcomes will be summarised with a relative risk (RR) and 95% confidence intervals (CIs) and compared between the two groups using a χ^2^ test (or Fisher’s exact test where applicable). Comparison of the number of patients with keloid formations within groups and between the two groups will be done using a χ^2^ test. A *t* test will be used to assess continuous non-skewed variables; these will be presented as means with standard deviations. For skewed ordinal data, the Mann–Whitney *U* test will be used; these data will be presented as medians with interquartile ranges. Difference in the effect over the sub-groups will be assessed using logistic regression. Outcomes of interest will be number of patients with keloid formation, changes in the appearance and specification of the keloid scar, depression, anxiety, stress and patient’s satisfaction. Changes in the appearance and specification of the keloid scar after the intervention will be summarised into two groups (binary outcome: 0 = changed, 1 = not changed) and will be analysed using logistic regression adjusting for the number of keloids formed and demographic variables. A *p* < 0.05 will be considered statistically significant.

### Data and safety monitoring

The patients will be reviewed on the ward post caesarean section for any signs of complications until discharge. The patients will be given a phone number to call to contact the investigator in case of any reaction and complication in relation to the caesarean section scar. Advice will be given to patients over the phone, or they will be invited to present to hospital for review for further management.

There will be an independent data and safety monitoring committee, including assessors independent of the Department of Women’s and Newborn Health, who will review all data and conduct audits of the trial at intervals to assess the safety and critical efficacy of the intervention, disentangle harms of corticosteroids from surgical complications and accordingly decide whether to continue, modify or stop the clinical trial.

### Interim analysis

An interim analysis will be performed on the primary outcome of keloid formation in patients when enrolling 20 patients, including both the control and intervention groups, by a single statistician who is blinded to allocation and reports the results to the data and safety monitoring committee.

### Harms

If there is a reasonable suspected causal relationship with the intervention, the study researcher who is blinded to the allocations will report to the data and safety monitoring committee, who will in turn report the adverse events to the ethics committee to guarantee the safety of the patients. We do not expect any risks for either group (control or intervention).

### Confidentiality

The electronic data obtained from participants will be stored in a dedicated file in the computer of the Chief Investigator at Westmead Hospital. The computer will be secured with automatic screen locking after 5 min of inactivity, and no one except the research team will have access to the data. A specific password will be required to access the file containing data, and this file will also be protected against viruses or malicious software.

Participants’ personal identities will not be disclosed to anyone else or in publications or presentations. Only aggregated results will be reported, in which no individual or identifying data will be included.

The data will be disposed of 10 years after publication of the results as required by Western Sydney Local Health District (WSLHD) Human Research Ethics Committee (HREC) policy. After the retention period, the files containing data in addition to any backups will be sanitised, and the manager of WSLHD HREC will be notified at the completion of the disposal.

### Ancillary and post-trial care

After completing the trial, we will continue to evaluate and treat the patients in the future if they wish us to do so.

### Dissemination policy

The final results of the trial are planned to be published in a scientific journal and presented at medical conferences. We will follow the Consolidated Standards of Reporting Trials (CONSORT) statement guidelines updated in 2010 (http://www.consort-statement.org).

## Discussion

Keloid formation often can be prevented or reduced if anticipated with immediate therapy. Once established, keloids are more difficult to treat, with a high recurrence rate regardless of treatment. Corticosteroids are a commonly used and effective treatment for established keloids on wounds in other area [16–18]. Corticosteroids suppress inflammation by inhibiting fibroblasts and mitosis while increasing vasoconstriction in the scar [19, 20]. Their effectiveness and side effects in subsequent caesarean section for those with established keloids has not been established. Yet caesarean section is easily the most common operation done, and keloid formation in these young women of childbearing age can be very distressing [3, 6, 21–23].

Other treatment modalities have also been used for keloid scars after caesarean section. Radiation therapy has been used with some clinical success [24, 25]. However, this modality of treatment requires multiple visits for radiation therapy during the post-natal period when more time is needed to care for the newborn. Our mode of treatment with a single administration of triamcinolone acetonide takes into consideration the need of the mother to have time to care for her newborn.

Common adverse effects of triamcinolone acetonide include atrophy, telangiectasia and hypopigmentation [26]. Steroids have been implicated as an aetiology in delayed wound healing. Although there is evidence in the literature that steroids delay wound healing, most studies are performed in vitro or use high systemic doses. Although wound disruptions have occurred in patients taking corticosteroids [26], treatment doses are generally below the level required for inhibition of wound healing in clinical practice. Acute, high-dose systemic corticosteroid use likely has no clinically significant effect on wound healing [27]. There is a retrospective evaluation study of post-operative intralesion steroid injections on wound healing [28]. Overall, there was not a statistically significant difference between the steroid groups and the non-steroid group. Therefore, one-time post-operative intralesion steroid injections were not found to delay wound healing [28].

Local infection was reported in an earlier study after the sub-dermal corticosteroid injection therapy [29]. However, this has not been reported in a more recent research in which they injected 40 mg/ml triamcinolone acetonide (0.1 ml) sub-dermally [30]. This risk has been suggested to increase after an overdose of the local injection of corticosteroid [29]. Since we aim to use a small dose of triamcinolone acetonide (10 mg/1 ml) along the entire length of each edge of the skin incision at the time of caesarean section, we expect this risk to be minimal. Nevertheless, we will carefully assess the participants in our study to ensure safety.

We noted the established use of triamcinolone acetonide suspension 10–40 mg/ml in the treatment of other surgical wounds, where it is injected sub-dermally depending on the site. This treatment will eventually flatten 50– 100% of keloids, with a 9–50% recurrence rate [10, 31]. The use of corticosteroid injection following keloid surgery reduces the recurrence rate to less than 50% [32]. Usually, two or three injections are given a month apart; however, therapy can continue for 6 months or longer [33].

New keloids are more responsive to therapy than older, established lesions. In theory, this injection will prevent any scar formation before treatment is started. If our study hypotheses are true, then steroid injection presents as a safe and sustainable treatment in the management of keloid scars. Our findings will be particularly useful for patients unable to undergo cosmetic surgery due to clinical or financial reasons and in under-resourced settings both within Australia and internationally. Since we do not have access to the plastic surgery data, we are unable to investigate the cost-effectiveness of the treatment. However, we hope that our findings will provide knowledge for further future research investigating the cost-effectiveness of sub-dermal triamcinolone acetonide for the treatment of caesarean section keloid scars.

### Trial status

The present protocol is version number 4, dated October 2018. The recruitment began in May 2019 and is expected to complete by June 2021.

## Additional file

Additional file 1: SPIRIT checklist: recommended items to address in a clinical trial protocol and related documents. (DOC 135 kb)

## Data Availability

Not applicable.
